# Predicting the network of substrate-enzyme-product triads by combining compound similarity and functional domain composition

**DOI:** 10.1186/1471-2105-11-293

**Published:** 2010-05-31

**Authors:** Lei Chen, Kai-Yan Feng, Yu-Dong Cai, Kuo-Chen Chou, Hai-Peng Li

**Affiliations:** 1 Institute of Systems Biology, Shanghai University, Shanghai 200444, PR China; 2 College of Information Engineering, Shanghai Maritime University, Shanghai 201306, PR China; 3 Centre for Computational Systems Biology, Fudan University, Shanghai 200433, PR China; 4 Division of Imaging Science, Medical School, Stopford Building, The University of Manchester, M13 9PT, UK; 5 Gordon Life Science Institute, San Diego, California 92130, USA; 6 CAS-MPG Partner Institute for Computational Biology, Shanghai Institutes for Biological Sciences, Chinese Academy of Sciences, Shanghai 200031, PR China

## Abstract

**Background:**

Metabolic pathway is a highly regulated network consisting of many metabolic reactions involving substrates, enzymes, and products, where substrates can be transformed into products with particular catalytic enzymes. Since experimental determination of the network of substrate-enzyme-product triad (whether the substrate can be transformed into the product with a given enzyme) is both time-consuming and expensive, it would be very useful to develop a computational approach for predicting the network of substrate-enzyme-product triads.

**Results:**

A mathematical model for predicting the network of substrate-enzyme-product triads was developed. Meanwhile, a benchmark dataset was constructed that contains 744,192 substrate-enzyme-product triads, of which 14,592 are networking triads, and 729,600 are non-networking triads; i.e., the number of the negative triads was about 50 times the number of the positive triads. The molecular graph was introduced to calculate the similarity between the substrate compounds and between the product compounds, while the functional domain composition was introduced to calculate the similarity between enzyme molecules. The nearest neighbour algorithm was utilized as a prediction engine, in which a novel metric was introduced to measure the "nearness" between triads. To train and test the prediction engine, one tenth of the positive triads and one tenth of the negative triads were randomly picked from the benchmark dataset as the testing samples, while the remaining were used to train the prediction model. It was observed that the overall success rate in predicting the network for the testing samples was 98.71%, with 95.41% success rate for the 1,460 testing networking triads and 98.77% for the 72,960 testing non-networking triads.

**Conclusions:**

It is quite promising and encouraged to use the molecular graph to calculate the similarity between compounds and use the functional domain composition to calculate the similarity between enzymes for studying the substrate-enzyme-product network system. The software is available upon request.

## Background

Metabolism (the Greek word for "change" or "overthrow") is the biochemical modification of chemical compounds in living organisms and cells. It comprises a series of chemical reactions that occur in a cell and enable it to keep living, growing and dividing. Without metabolism we would not be able to survive. Metabolism comprises a series of chemical reactions that occur in a cell and enable it to keep living, growing and dividing. Metabolism usually consists of sequences of enzymatic steps, the so-called metabolic pathways. The number of metabolic pathways is very large, reflecting the fact that "life is extremely complicated". Metabolic pathways interact in a complex way in order to allow an adequate regulation. This interaction includes the enzymatic control and hormone control. In the current study, we are focused on the enzyme control category, where metabolic pathway is the network linking various chemical reactions of compounds (substrates or products) catalyzed by enzymes. As is known, many metabolic pathways are available in the pathway databases, such as KEGG PATHWAY [1], which enable us to analyze known metabolic pathways. However, since there are many compounds and enzymes whose biological functions are not discovered completely, many reactions cannot be determined. Thus, determination of the network of substrate-enzyme-product triads (whether the substrate can be transformed into the product with the catalyst enzyme) would be very helpful for expanding our knowledge about the metabolic pathways, and conducting in-depth studies in this regard. However, it is time-consuming and expensive to determine the network through biological experiments alone. Therefore, it is highly desired if an automated method can be developed to address this problem. Encouraged by the successes of using computational approaches to tackle various problems in different biological systems (see, e.g., [2-7]), here we are to develop a different computational approach for predicting the network of substrate-enzyme-product triads.

The benchmark dataset used in this study consists of positive triads and negative triads, where the number of negative triads was about 50 times as many as positive ones. To evaluate the prediction model, one-tenth triads were randomly selected as testing samples and the rest triads used to train the prediction engine. The Nearest Neighbour Algorithm [8,9] was used to conduct prediction, where the metric to measure the nearness was formulated by combining the compound similarity and functional domain composition. The compound similarity was calculated based on the SMILES [10,11] and graph representations [12]; while the functional domain composition representations [13,14] were used to represent the enzyme samples and estimate their similarity. The highest accuracy thus obtained in predicting the positive triads was 95.41%. Interestingly, it was observed through this research that similar triads always tended to have the same network.

## Methods

### Materials

Molecular samples were downloaded from the public database KEGG [15,16] at http://www.genome.jp/kegg/ (release 53.0 in 2010), from which 16,144 molecules were retrieved. Among these molecules, only 2123 compounds take part in the main reactant-pairs in each metabolic reaction of yeast. For these selected small molecules, after removing those that had no information to calculate their similarity with other small molecules, we had 1,326 small molecules left; for enzyme molecules, after removing those whose functional domain compositions were not available, 939 enzyme molecules of yeast genome were obtained.

Although a same substrate might be converted into many products with different catalyst enzymes, a triad and its network would be unique. Each of the triads in the positive dataset consists of two small molecules (one for the substrate and one for the product) and one enzyme molecule. All the triads in the positive dataset were determined by solid experiments, and they were extracted from two KEGG files "reaction" and "enzyme", downloaded from ftp://ftp.genome.jp/pub/kegg/pathway/map/ (8th January, 2010). Each of the samples in the negative dataset, the so-called "negative triad", was generated by randomly picking two small molecules (one for the substrate and one for the product) and one enzyme molecule. Since the possibility for such three molecules to be a positive triad was extremely low, the credibility of the negative dataset thus constructed would be also very high. Also, to reflect the real world that the number of positive triads is much less than that of the negative ones, the negative triads were generated 50 times as many as the positive ones. The final benchmark dataset thus constructed contains 14,592 positive triads and 729,600 negative triads. Positive triads are also termed as networking triads, and negative triads termed as non-networking triads.

In order to evaluate the prediction model, one-tenth positive triads and one-tenth negative triads were randomly selected as testing samples, while the rest triads in the benchmark dataset were used to train the prediction engine. The detail information for the (1,460+72,960) = 74,420 testing samples and (13,132+656,640) = 669,772 training samples can be found in Additional File 1.

### Encoding Methods

A key step for conducting accurate prediction and analysis is to effectively encode and compare the three components: substrates, enzymes, and products. Since substrates and products are compounds, some established methods, such as SMILES [10,11] and MACCS keys [17,18] can be used to estimate the similarity of compounds. Recently, a method based on graph theory was proposed to measure the similarity of two compounds by means of the undirected graph [12]. Using graphic approaches to study biological systems can provide an intuitive vision and useful insights for helping analyze complicated relations therein, as indicated by many previous studies on a series of important biological topics, such as enzyme-catalyzed reactions [19-26], protein folding kinetics and folding rates [27-29], inhibition of HIV-1 reverse transcriptase [30-32], inhibition kinetics of processive nucleic acid polymerases and nucleases [33], and drug metabolism systems [34]. In this study, a different graph approach [12] will be utilized as described below.

#### Graph representation

Using graph representation to estimate the similarity of two compounds was proposed by Hattori et al. [12]. According to their method, each chemical structure can be represented by a two-dimensional (2D) graph where the vertices correspond to the atoms and the edges correspond to the bonds between them. The similarity of the two compounds is estimated by detecting their common subgraphs, followed by aligning them accordingly. The similarity score between two compounds by the graph representation can be calculated by the online web-server at http://www.genome.jp/ligand-bin/search_compound. However, the web-server only provides similarity scores that are greater than 0.4. Accordingly, in the current study, the similarity of two compounds is assigned to be zero if it is less than 0.4. The similarity score thus obtained between two compounds *c*_1 _and *c*_2 _is denoted by *S*_graph_(*c*_1 _*c*_2_).

Meanwhile, the following non-graphic SMILES [10,11] approach will also be utilized to facilitate comparison.

#### SMILES

Abbreviated from the full name of "Simplified Molecular Input Line Entry System" [10,11], SMILES is a line representation for compound, which consists of a series of characters without including spaces. The similarity score between two compounds with the SMILES representation can be obtained from a pre-computed database called STITCH [35] at http://stitch.embl.de/cgi/, where the similarity score between two compounds *c*_1 _and *c*_2 _is denoted by *S*_SMILES_(*c*_1_, *c*_2_)/1000. The developers of STITCH applied the open-source Chemistry Development Kit [36] to calculate the chemical fingerprints and used the Tanimoto 2 D chemical similarity scores [37,38].

#### Functional domain composition representation

Since enzyme belongs to protein, we can use various descriptors for proteins as summarized in a recent review [39] to represent enzymes. In this study, we adopted the functional domain composition to represent the enzyme samples because it has been successfully used for predicting various protein attributes [6,13,14,40-46]. The concept of protein functional domain composition was first introduced by Chou and Cai for predicting protein subcellular localization [13], where the SBASE-A database [47] was used that contained 2,005 functional domains. In this research, we used a more complete database, the InterPro database (release 23.1, December 2009) [48] that contained 21,144 functional domain entries. Accordingly, by following the similar procedures as elaborated in [13], an enzyme molecule *e *can be formulated as the following 21144-D vector(1)

where *x*_*i *_= 1 if there is a hit at the *i*-th functional domain entry by searching the InterPro database for the enzyme sample *e*; otherwise, *x*_*i *_= 0. Thus, the similarity between two enzyme molecules, *e*_1 _and *e*_2 _is given by [13](2)

where  is the dot product of two vectors, and  and  are their modulus, respectively.

Thus, the similarities between any two substrate-enzyme-product triads can be calculated using the above equations, as will be further discussed below.

### K-Nearest Neighbour Algorithm (KNN)

In this research, the K-Nearest Neighbour (KNN) algorithm [5,8] was applied to predict a query triad belonging to networking or non-networking. To utilizing the KNN algorithm, we have to first define a metric to measure the nearness between two triads *T*_1 _= (*s*_1_, *e*_1_, *p*_1_) and *T*_2 _= (*s*_2_, *e*_2_, *p*_2_), where *s*_1_, *e*_1_, *p*_1 _represent the substrate, enzyme, product in the first triad *T*_1_, and *s*_2_. *e*_2_, *p*_2 _those in the second triad *T*_2_. Since there are three members in each triad, and we do not know which one of the three will play more important role in determining the network, let us first define the following metric with a weight parameter to measure the nearness between the two triads:(3)

where the weight factor *w *can be obtained by optimizing the predicted result. According to the KNN rule [8,49,50], also named the "voting KNN rule", a query triad should be assigned to the class represented by a majority of its *K *nearest neighbours. If the majority of its K nearest neighbour triads belong to the triad networking, and so does the query triad; otherwise, it belongs to the non-networking triad.

### Accuracy Measurement

The accuracy of prediction is defined by(4)

where TP represents true positives, TN true negative, FP false positives, and FN false negative [51-54], with(5)

for the sensitivity and(6)

for the specificity.

In order to evaluate the performance of prediction models more accurate, Matthew's correlation coefficient (MCC) [55] was employed in this study, which is defined by(7)

## Results

The predicted accuracies with *K *= 1 and *w *= 1/4, 1/2, and 3/4 for the testing triads in which the substrate and product compounds were represented by SMILES are given in Table 1, while those with graph to represent the compounds are given in Table 2. The detailed predicted results are provided in Additional File 2.

**Table 1:** Prediction accuracies of testing samples using SMILES to represent substrate and product compounds.

*w*	Prediction accuracy for each class (%)		Overall prediction accuracy (ACC) (%)	Matthew's correlation coefficient (MCC) (%)
			
	Networking triads (SN)	Non-networking triads (SP)		
1/4	94.25	94.95	94.94	49.14
1/2	83.01	87.77	87.68	28.62
3/4	79.11	83.74	83.65	22.94

**Table 2:** Prediction accuracies of testing samples using graph to represent substrate and product compounds.

*w*	Prediction accuracy for each class (%)		Overall prediction accuracy (ACC) (%)	Matthew's correlation coefficient (MCC) (%)
			
	Networking triads (SN)	Non-networking triads (SP)		
1/4	95.41	98.77	98.71	75.67
1/2	85.68	97.56	97.32	58.39
3/4	82.19	97.47	97.17	55.77

It can be seen from Table 1 and 2 that, when *w *= 1/4 and using the graph representation for the substrate and product compounds, we obtained not only the highest overall prediction accuracy (ACC = 98.71%) but also the highest MCC value (MCC = 75.67%), indicating that the graph representation approach is really quite effective.

Shown in Table 3 are the prediction accuracies when *K *= 3, 5, and *w *= 1/4. Compared with the case of *K *= 1, although the rate for the non-networking triads was remarkably increased somewhat, the rate for the networking triads was decreased.

**Table 3:** Prediction accuracies of testing samples using different K.

Representation of compound	*K*	Prediction accuracy for each class (%)	
		
		Networking triads (SN)	Non-networking triads (SP)
SMILES	3	92.67	92.03
	5	89.79	92.92

Graph	3	95.34	99.48
	5	94.18	99.48

## Discussion

Our results have shown that, in the study of the substrate-enzyme-product triad network, it is quite promising and encouraged to use the functional domain composition to represent enzyme and use the graph descriptor to represent substrate and product compounds, fully consistent with the advantage of using functional domain to represent enzyme samples for predicting enzyme family classification [56-58] and the advantage of using the graph descriptor to represent compounds as discussed in [12].

**Figure 1:**
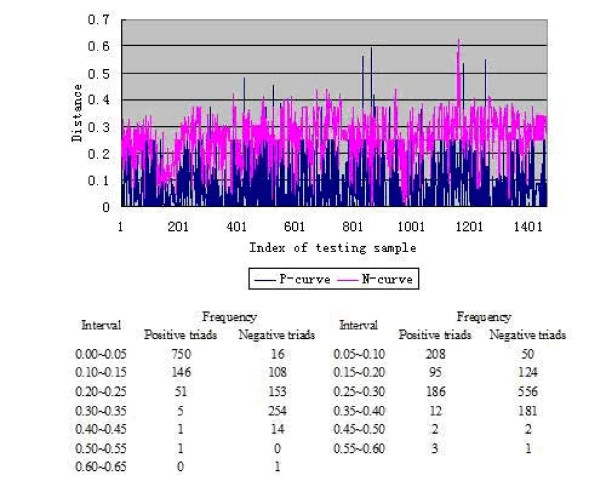
**P-curve and N-curve**.

As indicated in Additional File 1, there are 1,460 positive triads in testing samples. For each of these positive triads *T*_*i *_(*i *= 1,2,⋯,1460), we calculated the distance of Eq.3 (with *w *= 1/4 and using the graph descriptor for substrate and product compounds) from *T_i _*to its nearest positive triad and nearest negative triad in the training set, respectively. Denote the two distances thus obtained by *P_i _*and *N_i_*, respectively. Shown in Fig 1 are two curves generated from *P_i _*and *N_i_*, named as P-curve and N-curve, respectively. The P-curve is the one with the index *i *of *T_i _*as its X-axis and *P_i _*as its Y-axis. The N-curve is the one with the index *i *of *T_i _*as its X-axis and *N_i _*as its Y-axis. It can be seen from Fig 1 that the N-curve is almost always above the P-curve, meaning that the distances of the 1,460 testing triads to their nearest positive triads in the training set are almost always smaller than those to their nearest negative triads in the training set, fully consistent with the very high success rate of 95.41% for predicting the 1,460 networking triads, as shown in Table 2. Furthermore, for the distribution of these distance values, there are 1,104 (75.62%) *T_i _*with *P_i _*< 0.15, while there are only 174 (11.92%) *T_i _*with *N_i _*< 0.15. The most of *N_i _*(1268, 86.85%) were clustered in the interval from 0.15 to 0.4, indicating that the distance defined by Eq.3 for the KNN algorithm with *w *= 1/4 can separate the positive triads and negative triads very well. Also, since the distance of Eq.3 is defined based on the similarities of two substrates, two enzymes and two products, the smaller the distance between the two triads, the more similar the two triads are. It is interesting to see from the current study that the similar triads as defined by our formulation almost always exhibit the same network.

**Figure 2:**
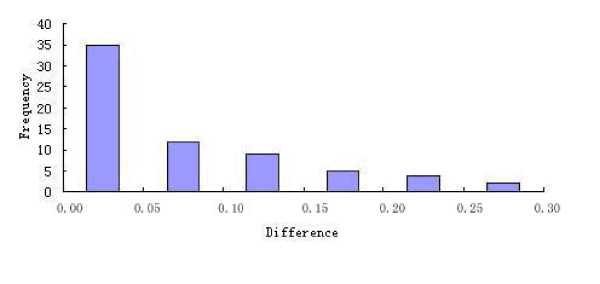
**Distribution of differences in Table 4**.

As indicated by comparing the results in Table 1, Table 2 and Table 3, the best predicted rate for the 1,460 networking triads in the testing set was 95.41%, with *w *= 1/4 and *K *= 1. Of these triads, 67 were mispredicted. It is instructive to see the reason behind these by examining Table 4, where the difference between the distance to the nearest positive triad and the distance to the nearest negative triad for each of the 67 misclassified triad samples was given. As we can see from the table, the maximum difference was 0.285 and the minimum difference was 0.000256. Shown in Fig 2 is the distribution of the distance differences listed in Table 4. Of the 67 misclassified positive samples, 47 (70.15%) samples are with the distance differences less than 0.1, implying that the mispredicted triads are pretty close to the margin of correct prediction, and that the current metric as defined in Eq.3 for measuring the nearness for the KNN algorithm is quite effective.

**Table 4:** Distance to nearest positive triads and negative triads of misclassified positive triads.

Substrates	Enzymes	Products	Distance		Differences
				
			Positive triads	Negative triads	
C00002	YIL139C	C06397	0.24	0.19125	0.04875

C00002	YPL271W	C00008	0.25	0.22125	0.02875

C00002	YPR033C	C00020	0.1	0.03375	0.06625

C00003	YKR066C	C00004	0.25	0.225	0.025

C00003	YPR167C	C00004	0.25	0.177831	0.072169

C00010	YER090W	C00024	0.25	0.1125	0.1375

C00010	YER178W	C00024	0.189188	0.1425	0.046688

C00024	YAL054C	C00033	0.21	0.199626	0.010374

C00024	YCL030C	C06548	0.25	0.0975	0.1525

C00024	YLR153C	C00033	0.21	0.199626	0.010374

C00025	YHR037W	C03912	0.375	0.202643	0.172357

C00026	YIR034C	C00449	0.271688	0.25	0.021688

C00035	YGL047W	C00096	0.1875	0.165	0.0225

C00037	YOL049W	C00051	0.48375	0.25	0.23375

C00047	YPL096W	C12989	0.25	0.225	0.025

C00055	YBL013W	C04121	0.177831	0.12375	0.054081

C00055	YDR410C	C04121	0.25	0.22125	0.02875

C00055	YKR069W	C04121	0.25	0.19125	0.05875

C00065	YBR263W	C00143	0.375	0.25	0.125

C00065	YLR058C	C00143	0.375	0.25	0.125

C00083	YPL231W	C12647	0.073223	0.02625	0.046973

C00085	YKL104C	C00352	0.25	0.2325	0.0175

C00086	YIR029W	C00499	0.4525	0.375	0.0775

C00096	YBR252W	C00144	0.25	0.12	0.13

C00096	YGR036C	C00636	0.25	0.24375	0.00625

C00108	YDR354W	C04302	0.375	0.2325	0.1425

C00109	YCL018W	C06032	0.383376	0.36625	0.017126

C00118	YGL026C	C03506	0.375	0.37375	0.00125

C00143	YGL125W	C00440	0.3025	0.28375	0.01875

C00155	YNL256W	C01118	0.25	0.22125	0.02875

C00167	YJR131W	C00191	0.25	0.21	0.04

C00191	YOR065W	C05787	0.25	0.19875	0.05125

C00223	YDR062W	C12096	0.0825	0.082244	0.000256

C00223	YMR296C	C12096	0.0825	0.04875	0.03375

C00234	YDR408C	C04376	0.36625	0.32125	0.045

C00333	YJR153W	C00470	0.375	0.12375	0.25125

C00448	YDL205C	C16144	0.225	0.19125	0.03375

C00582	YHL003C	C05598	0.25	0.1875	0.0625

C00582	YKL008C	C05598	0.25	0.1875	0.0625

C00632	YDR120C	C05831	0.25	0.15	0.1

C00652	YML086C	C06316	0.565	0.36625	0.19875

C00842	YDR127W	C06017	0.1125	0.09	0.0225

C00864	YDR531W	C03492	0.25125	0.25	0.00125

C00931	YDL205C	C01024	0.59625	0.375	0.22125

C01063	YBL015W	C09813	0.1275	0.1125	0.015

C01079	YDR044W	C03263	0.41875	0.25	0.16875

C01096	YCL030C	C02888	0.25	0.22875	0.02125

C01100	YIL116W	C01267	0.375	0.25	0.125

C01902	YML008C	C08830	0.375	0.25	0.125

C02411	YGR155W	C03058	0.09	0.075	0.015

C02909	YHR007C	C14098	0.25	0.195	0.055

C03012	YDR402C	C11713	0.36375	0.2575	0.10625

C03598	YPR167C	C04297	0.25	0.1875	0.0625

C04751	YAR015W	C04823	0.34	0.32875	0.01125

C04874	YDR452W	C05925	0.16875	0.125	0.04375

C06102	YLR231C	C06105	0.535	0.25	0.285

C06397	YBR029C	C07838	0.18375	0.17625	0.0075

C06599	YNL202W	C06600	0.147938	0.113376	0.034562

C06714	YDR127W	C06723	0.0975	0.08625	0.01125

C07649	YDR402C	C12673	0.55	0.3625	0.1875

C07732	YGR234W	C07733	0.3075	0.25	0.0575

C09811	YGL063W	C09812	0.1125	0.10125	0.01125

C11907	YPR118W	C11908	0.25	0.22875	0.02125

C11923	YFR015C	C12384	0.25	0.03	0.22

C11923	YLR258W	C12384	0.25	0.03	0.22

C14082	YHR007C	C14089	0.25	0.195	0.055

C15786	YGR060W	C15797	0.09375	0.08625	0.0075

Like most of the other prediction methods, the current prediction method also has its own limitation. For example, for those query triads without any similarity at all to any of the triads in the training datasets, the performance of the current prediction method might be poor. This is because the current prediction method was established on the basis of the "triad similarity", i.e., the similarity between substrates, between enzymes, and between products.

As pointed out by one of the anonymous reviewers, it would be interesting to further discuss the current algorithm from the viewpoint of divergent and convergent evolution [59]. We shall work on such an interesting topic in our future work.

## Conclusions

Metabolic pathway is one of the key biological networks, consisting of many metabolic reactions involving substrates, enzymes, and products, where substrates can be transformed into products with some particular catalytic enzymes. Knowledge about the network of substrate-enzyme-product triads is very useful for in-depth studies of the metabolic pathways. It is both time-consuming and costly to determine the network through biological experiments alone, and hence it is highly desired to develop computational methods in this regard. The computational method reported in this paper can be used to identify the network of substrate-enzyme-product triads with quite high success rate. It is anticipated that the method may become a very useful tool for studying drug metabolism systems. Meanwhile, as shown through this study, it is quite promising to introduce the molecular graph and functional domain composition into this area. Since user-friendly and publicly accessible web-servers represent the future direction for developing practically more useful predictors [60], we shall design a user-friendly web-server for the prediction method so that many experimental bench scientists can easily use it to get the desired results without the need to go through all the mathematical details.

## Authors' contributions

LC, KYF, and YDC did materials preparation, method design and programming. LC wrote the paper, KYF, YDC, KCC, and HPL gave scientific advice and made revision. All authors have read and approved the final manuscript.

## Supplementary Material

Additional file 1 **Networking and non-networking triad samples in the training dataset and testing dataset used in this study**. Each triad consists of a substrate, an enzyme, and a product. Click here for file

Additional file 2 **The detailed prediction results**. This file lists the prediction results for each of the testing sample in Additional File 1. Click here for file
